# Viscoelastic point-of-care testing for monitoring unfractionated heparin in critically ill patients: a prospective observational study comparing ClotPro® IN/HI-Test, aPTT and Anti-Xa activity

**DOI:** 10.1186/s12871-026-03781-4

**Published:** 2026-03-24

**Authors:** Martin Mirus, Anna Dietze, Oliver Tiebel, Jan Beyer-Westendorf, Oliver Grottke, Falko Tesch, Peter Markus Spieth, Lars Heubner

**Affiliations:** 1 Department of Anesthesiology and Intensive Care Medicine, University Hospital “Carl Gustav Carus”, Technische Universität Dresden, Fetscherstraße 74, Dresden, 01307 Germany; 2 Institute of Clinical Chemistry, University Hospital Carl Gustav Carus, Technische Universität Dresden, Dresden, Germany; 3 Division of Thrombosis and Haemostasis, Department of Medicine I, University Hospital Carl Gustav Carus, Technische Universität Dresden, Dresden, Germany; 4 Department of Anesthesiology, University Hospital Aachen, RWTH, Aachen University, Aachen, Germany; 5 Centre for Evidence-Based Healthcare (ZEGV), University Hospital and Faculty of Medicine Carl Gustav Carus, Technische Universität Dresden, Dresden, Germany

**Keywords:** Unfractionated heparin, Anticoagulation monitoring, Anti-factor Xa, Viscoelastic testing, Intensive care, Point-of-care testing

## Abstract

**Background:**

Monitoring of unfractionated heparin (UFH) in critically ill patients is challenging due to disease-related alterations of the coagulation system, directly affecting conventional laboratory tests, including activated partial thromboplastin time (aPTT). Measurement of anti-factor Xa activity (Anti-Xa) as an alternative to aPTT often lacks 24/7 availability. Whole blood viscoelastic testing (VET) may provide a feasible point-of-care alternative to guide UFH dosing in this setting. Additionally, VET is readily available in the environment found in our clinic, The aim of this study was to evaluate the diagnostic performance of viscoelastic testing compared with activated partial thromboplastin time for monitoring unfractionated heparin in critically ill patients and to define clinically applicable viscoelastic thresholds using anti-factor Xa activity as the reference standard.

**Methods:**

In this prospective observational study for UFH monitoring Anti-Xa, aPTT, and the VET clotting time IN/HI ratio (ClotPro®; Haemonetics, Boston, MA, USA) were determined in simultaneously taken blood samples. Anti-Xa results were used as the reference standard to categorize ongoing UFH anticoagulation intensity into four categories (prophylactic, sub-therapeutic, therapeutic, and overdosed). Diagnostic performance of aPTT and CT IN/HI ratio were evaluated against Anti-Xa using ROC analysis and Cohen’s Kappa. Test performance was evaluated using ROC analysis, multiclass classification, and weighted Cohen’s kappa.

**Results:**

Between September 2020 and July 2022, 466 datasets from 75 ICU patients receiving intravenous UFH were analyzed. Compared to aPTT, AUC values for CT IN/HI were higher for detecting prophylactic (0.817 vs. 0.726), therapeutic (0.690 vs. 0.494) and overdosed (0.845 vs. 0.708) UFH levels. Thresholds of > 1.4, > 1.9, and > 2.3 for the CT IN/HI ratio corresponded to sub-therapeutic, therapeutic, and overdosed ranges, respectively. The CT IN/HI ratio showed moderate agreement with Anti-Xa classification (κ = 0.450), while aPTT agreement was insufficient (κ = 0.220).

**Conclusions:**

The CT IN/HI ratio offers a reliable point-of-care alternative to standard UFH monitoring, in the case it is readily available. It provides better alignment with Anti-Xa activity than aPTT and may improve anticoagulation management in critically ill patients, especially when Anti-Xa testing is delayed or unavailable.

**Trial registration:**

DRKS-ID DRKS00028689 (https://drks.de/search/de), PI: Lars Heubner, retrospectively registered 30.03.2022.

**Supplementary Information:**

The online version contains supplementary material available at 10.1186/s12871-026-03781-4.

## Introduction

Critically ill patients are at increased risk for both thromboembolic and bleeding complications [1–5]. Thromboprophylaxis is therefore mandatory in intensive care unit (ICU) patients, and many also require higher-intensity anticoagulation, either in therapeutic or intermediate dosing regimens. Unfractionated heparin (UFH) remains the agent of choice particularly in patients undergoing ECMO [6, 7] and in cases of severe renal impairment where certain low-molecular-weight heparins (LMWH) may be contraindicated or require dose adjustment, due to its short half-life and less renal excretion [8, 9]. Traditionally, UFH monitoring is based on activated partial thromboplastin time (aPTT) measurements despite well-documented limitations of this method in critically ill patients [10]. The aPTT reflects the intrinsic pathway of the classic coagulation cascade and its ability to produce thrombin [8]. Various clot-based and chromogenic assays are available for measuring aPTT [10–12]. Notably, aPTT measurement is significantly affected by the levels of coagulation factors such as XII, XI, IX, VIII, V, and X. The absence of only one single coagulation factor – hereditary or due to consumption – can result in a prolonged aPTT, even when other factors remain within normal ranges [7, 13–16]. At the same time, upregulation of FVIII, an acute phase protein which is often increased in ICU patients during trauma, infection, inflammation, or malignant diseases, can “stabilize” aPTT measurements even with supratherapeutic heparin dosages, leading to an in vitro ceiling effect of aPTT and an underestimation of in vivo anticoagulant intensity [13, 17–19]. Since critically ill patients frequently exhibit abnormal levels of coagulation factors, aPTT may not accurately represent the pharmacodynamic effect of UFHin this vulnerable population where exact assessments and adjustments of anticoagulant may be vital [13, 20–22].

Measurement of anti-factor Xa activity (Anti-Xa) [6, 13, 23–25] provides a more specific UFH assessment because it quantifies the heparin–antithrombin–mediated inhibition of factor Xa and is less susceptible to factor level variations. Although Anti-Xa assays are not without limitations – including calibration variability, effects of hyperbilirubinemia, hypertriglyceridemia or haemolysis and incomplete 24/7 availability in many institutions – they are increasingly recommended over aPTT for UFH monitoring in critically ill patients, as highlighted in recent consensus statements and reviews [26, 27]. These publications underscore that, despite practical and analytical constraints, Anti-Xa currently represents the most accurate and least confounded laboratory measure of UFH activity in this population. Accordingly, in the present study Anti-Xa was used as the reference standard.

Point-of-care (POC) methods for monitoring UFH offer a promising alternative. Among them, the activated clotting time (ACT) is widely used in ICUs and operating rooms due to its ease of use and rapid turnaround [28, 29]. However, the ACT has demonstrated limited sensitivity at prophylactic and intermediate UFH doses and is influenced by a variety of factors including temperature, antithrombin, hematocrit, platelet count, fibrinogen levels, and inflammation [30–32]. Viscoelastic testing (VET) presents another point-of-care diagnostic modality. While ACT and VET share the advantages of bedside applicability and rapid result availability, they differ in their measurement approach. ACT terminates at the time of clot detection, whereas VET provides a continuous graphical assessment of clot formation in whole blood. Recent developments have enabled the use of VET for monitoring UFH through paired intrinsic (IN) and heparinase (HI) assays, allowing the calculation of a CT ratio of both tests that may reflect the specific contribution of UFH to the overall anticoagulant effect [33, 34]. The present prospective study aimed to evaluate the performance of the CT IN/HI ratio from point-of-care viscoelastic ClotPro® testing and compare it to both standard monitoring methods aPTT and Anti-Xa for assessing UFH anticoagulation in critically ill patients. Anti-Xa activity served as the reference standard because it directly reflects heparin’s inhibitory effect on factor Xa and is widely regarded as the most reliable measure of UFH activity in this population. Furthermore, the study sought to identify a clinically applicable threshold for the CT IN/HI ratio to guide appropriate UFH dosing in routine clinical practice for thromboprophylaxis or anticoagulation in critically ill patients.

### Objectives and outcomes

The primary objective of this study was to compare the diagnostic performance of the CT IN/HI ratio obtained from the ClotPro® VET device and the aPTT against Anti-Xa activity, which served as the analytical reference standard.

Specifically, the study aimed to determine the agreement of CT IN/HI and aPTT with Anti-Xa-defined anticoagulation intensity categories and to assess their ability to identify UFH underdosing and overdosing. These Anti-Xa–based intensity categories reflect real-world dosing targets in our ICU and were therefore used as the clinical reference for evaluating agreement, classification accuracy, and detection of under- and overdosing.

Secondary objectives included the identification of clinically applicable CT IN/HI thresholds corresponding to prophylactic, therapeutic, and supratherapeutic anticoagulation ranges, as well as the evaluation of their sensitivity, specificity, and predictive values. The study further sought to explore the suitability of the CT IN/HI ratio for multiclass classification of anticoagulation states and its potential pragmatic value as a bedside monitoring tool in settings where Anti-Xa testing is unavailable or delayed.

## Methods

### Ethics

The study was designed in accordance with the Declaration of Helsinki. Ethical approval was obtained from the Dresden University Ethics Committee (BO-EK-374072021). The requirement for informed consent was waived by the Dresden University Ethics Committee due to the observational design and use of de-identified data. The study was retrospectively registered in the German Clinical Trials Register (DRKS00028689). Study conduct and reporting followed the Strengthening the Reporting of Observational Studies in Epidemiology (STROBE) Statement guidelines [35].

### Study design and setting

This single-center, prospective observational clinical study was conducted in the ICU of the Department of Anaesthesiology and Intensive Care Medicine, University Hospital Carl Gustav Carus, Technische Universität Dresden, Germany. The study period spanned from September 2020 to July 2022. During this time, all critically ill patients receiving continuous intravenous UFH therapy within approved indications were consecutively enrolled. UFH dosing and monitoring were conducted in accordance with local institutional guidelines. UFH therapy is routinely assessed and dose-adjusted based on Anti-Xa activity, with Anti-Xa testing available 24 h a day. For dosing decisions, the following Anti-Xa target ranges are applied: prophylactic anticoagulation < 0.30 IU/mL, subtherapeutic anticoagulation 0.30–0.49 IU/mL, therapeutic anticoagulation 0.50–0.69 IU/mL, and supratherapeutic anticoagulation as levels ≥ 0.7 IU/mL [36, 37]. In our institution, continuous intravenous UFH is routinely used not only for therapeutic anticoagulation but also for thromboprophylaxis in critically ill patients. Consequently, Anti-Xa target ranges should reflect the intended anticoagulation intensity of the ongoing infusion (prophylactic, sub-therapeutic, therapeutic), rather than merely categorizing analytical results. This four-level classification is integral to daily UFH dose adjustment and directly guides clinical decision-making. The use of these predefined Anti-Xa ranges enabled a clinically relevant performance analysis of aPTT and the IN/HI ratio by assessing their ability to detect under-, on- and over-anticoagulation. Moreover, the multiclass structure permitted evaluation via ROC analysis, weighted κ, and multiclass classification metrics, which would not be feasible with a single dichotomous threshold. Anti-Xa levels for UFH monitoring are typically assessed every 6 to 8 h, with additional measurements performed as clinically indicated. If test values fall within the target range, UFH dosing is generally maintained unless the clinical context requires adjustment. In cases of out-of-range Anti-Xa levels, UFH doses are modified accordingly, and follow-up measurements are obtained within four hours to ensure appropriate anticoagulation.

### Anticoagulation monitoring

For the purpose of this study, all measurements of Anti-Xa levels, performed in daily clinical care routine, were complemented with simultaneous measurements of aPTT and VET clotting time IN/HI ratio (CT IN/HI) to enable direct comparison of these three monitoring strategies.

Analyses were conducted on STA-R Max 3 analyzers (STAGO Deutschland GmbH, Düsseldorf, Germany). For aPTT determination, the kaolin-activated STA®-C.K. PREST® reagent (Diagnostica Stago) was used. All blood samples for laboratory and VET testing were collected from an arterial catheter. Citrated samples were processed within two hours under standardized conditions. VET was performed using ClotPro® (Haemonetics, Boston, MA, USA), utilizing the intrinsic (IN) and heparinase (HI) assays. The ClotPro® device assesses clot formation by measuring the resistance of a blood sample in a rotating cylindrical cup. As clot firmness increases, cup rotation is increasingly impeded, and this mechanical signal is converted into a graphical representation of clot dynamics. Key parameters included: Clotting time (CT); Clot formation time (CFT); Maximum clot firmness (MCF); Maximum lysis (ML). The IN-test activates the intrinsic pathway using ellagic acid. The HI-test employs the same activation but incorporates heparinase to neutralize UFH activity. According to the manufacturer’s instructions for use, the heparinase contained in the HI-test neutralizes the anticoagulant effect of unfractionated heparin in the sample up to concentrations of approximately 7.5 IU/mL. At very high heparin concentrations a minor residual prolongation of clotting time may occur; however, manufacturer dose–response data indicate that CT prolongation remains below approximately 10% of baseline values for heparin concentrations up to 5 IU/mL. The diagnostic principle therefore relies on the comparison between the IN-test and HI-test clotting times to identify the heparin-specific contribution to coagulation inhibition. The CT IN/HI ratio, calculated by dividing CTIN by CTHI, serves as a surrogate marker for residual heparin effect. For ClotPro® analysis, 340 µL of citrated whole blood was pipetted into prefilled test cups, and assays were conducted at 37 °C over a 60-min runtime. No additional blood samples were collected for study purposes. All analyses were performed using residual blood from routine clinical laboratory samples obtained for standard anticoagulation monitoring. Therefore, no additional patient intervention was required.

### Data collection

Collected data included patient demographics, comorbidities, indications for UFH initiation, laboratory and viscoelastic coagulation parameters, and clinical outcomes. This design facilitated the evaluation of UFH safety and efficacy across various subpopulations of critically ill patients, particularly those with complex coagulopathy profiles or increased risk of thrombotic or haemorrhagic complications. For the purpose of this study, each measured Anti-Xa result was classified by measured Anti-Xa range: prophylactic, subtherapeutic, therapeutic, or supratherapeutic.

The observational nature of the study ensured that routine clinical management remained unaffected by protocolized interventions, thus providing real-world insight into UFH monitoring practices and outcomes in this challenging patient cohort.

### Statistical analysis

The diagnostic performance of the CT IN/HI ratio and aPTT was assessed based on their ability to identify corresponding Anti-Xa levels in matched samples. Anti-Xa served as the reference standard. Receiver operating characteristic (ROC) curves and the corresponding area under the curve (AUC) values were calculated for classification into prophylactic, therapeutic, and supratherapeutic categories. Interpretation of diagnostic performance according to AUC values: AUC > 0.9: excellent; AUC > 0.8: good: AUC > 0.7: Acceptable; AUC ≈ 0.5: No diagnostic value (random performance). The analyses assumed a prevalence of 20% prophylactic, 10% overdosed and, in the therapeutic group, a 50/50 split between sub-therapeutic and therapeutic anticoagulation. Relative weights for different types of misclassification were defined as follows: false-negative for prophylaxis = 2, for overdosing = 5, for subtherapeutic towards therapeutic = 1. Agreement between test classifications and Anti-Xa targets was further evaluated using weighted Cohen’s kappa with 95% confidence intervals. Differences between the areas under the ROC curves for aPTT and the CT IN/HI ratio were compared using DeLong’s test, a nonparametric method for statistically assessing whether two correlated ROC curves differ in diagnostic performance. Categorical variables are presented as absolute and relative frequencies. Continuous variables are reported as medians with interquartile ranges (IQR). All analyses were performed using R version 4.3.1 (R Foundation for Statistical Computing, Vienna, Austria). A data analysis and statistical plan was written, date-stamped, and recorded in the investigators’ files before data were accessed.

## Results

### Characteristics of the cohort

**Fig. 1:**
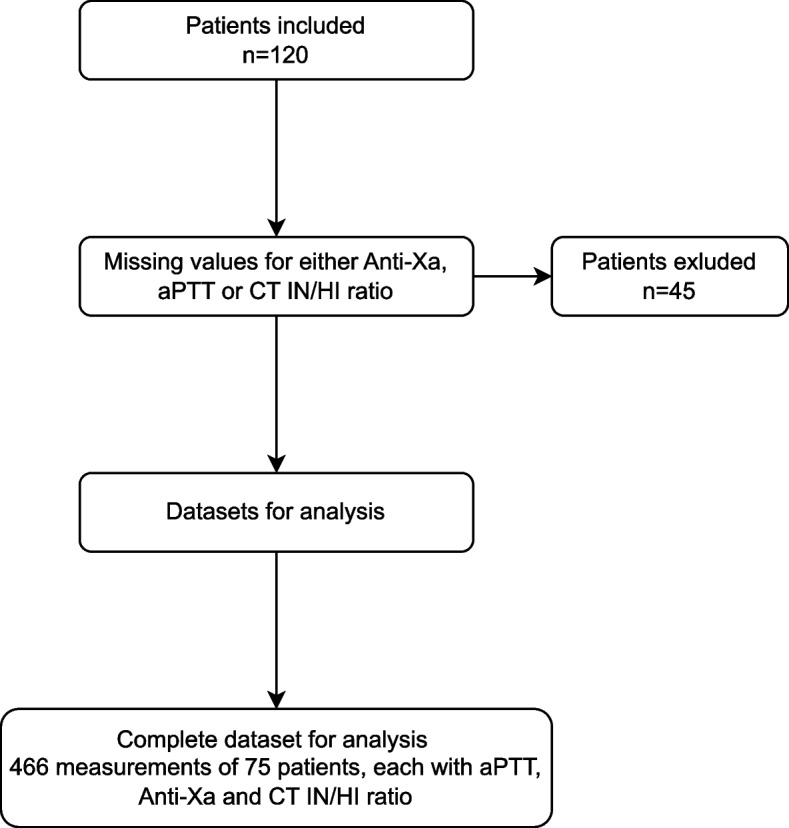
Study: flow chart. In this study, 120 patients with 735 datasets were enrolled. Due to incomplete datasets with measurements at the same time 45 patients were excluded. The remaining 75 patients with complete datasets, accounting for 466 measurements, were analyzed

For this study a total of 466 sets of blood samples from 75 patients were provided for matched-pair analyses (Fig. 1). The number of samples was not evenly distributed amongst patients due to their length of stay at the ICU and the exclution of tests due to measurement errors. The characteristics of the study cohort are summarized in Table 1. Results for groups based on clinically relevant Anti-Xa ranges are presented in Table 2.

**Table 1:** Characteristics of the study cohort

	*Study Cohort*
	*n* = 75
*General information*	
Age [years]	59 (52; 63)
Male Sex, n (%)	57 (76)
Body Mass Index [kg/m^2^]	31 (28; 34)
UFH dosage [IU · kg^−1^ · h^−1^]	17 (12; 23)
*Laboratory parameters*	
Fibrinogen [g/L]	5.3 (4.1; 6.6)
Platelet count [Gpt/L]	128 (80.3; 193)
Antithrombin [%]	77 (68; 86)
*Course of Disease*	
ECMO therapy, n (%)	55 (73.3)
Septic shock, n (%)	44 (58.6)
Thromboembolic Events*	*n* = 17
Pulmonary embolism, n (%)	5 (29.4)
Deep vein thrombosis, n (%)	7 (41.2)
Catheter-associated, n (%)	5 (29.4)
Bleeding Events*	*n* = 58
Bleeding BARC I, n (%)	25 (43.1)
Bleeding BARC II-IV, n (%)	32 (55.2)

Unless otherwise stated, values are presented as median (25th and 75th interquartile range)

*ECMO* Extracorporeal membrane oxygenation, *UFH* Unfractionated heparin

*after admission to our ICU and during anticoagulation therapy with UFH and anti-Xa target therapy monitoring

**Table 2:** Agreement of Viscoelastic Testing and aPTT with Anti-Factor Xa Categories for UFH Monitoring and the occurrence of bleeding events

Clinical classification of Anti-Xa test results	Prophylactic (Anti-Xa < 0.30 IU/ml)	Sub-therapeutic (Anti-Xa 0.30–0.50 IU/ml)	Therapeutic (Anti-Xa 0.50–0.70 IU/ml)	Overdosing (Anti-Xa ≥ 0.70 IU/ml)
*n* =	263	86	41	76
Anti-Xa [IU/ml]	< 0.3	0.3–0.5	0.5–0.7	> 0.7
aPTT [s]	43 (37; 53)	52 (46; 59)	52 (47; 56)	55 (50.5; 60)
CT IN/HI ratio	1.464 (1.169; 1.835)	1.843 (1.577; 2.232)	2.268 (1.882; 2.840)	2.726 (2.262; 3.195)
UFH dosing [IU · kg^−1^ · h^−1^]	15 (10; 19)	19 (16; 26)	21 (16; 26)	23 (18; 26)
Bleeding events	34 (12.9%)	13 (15.1%)	4 (9.8%)	7 (9.2%)

Measurement results for 75 patients with 466 sampling points with Anti-Xa values, aPTT, CT. aPTT, activated partial thromboplastin time; CT, clotting time; UFH, unfractionated heparin; IN, intrinsic test; EX, extrinsic test. Unless otherwise stated, values are presented as median (25th and 75th interquartile range)

Within the category Anti-Xa < 0.30 IU/ml, mean UFH dose was 15 IU/kg/h and the corresponding mean values for aPTT and CT IN/HI ratio were 43 s and 1.464, respectively.

Subtherapeutic Anti-Xa values (0.30–0.49 IU/ml) were achieved with a mean UFH dose of 19 IU/kg/h and the corresponding mean values for aPTT and CT IN/HI ratio were 52 s and 1.843, respectively.

A further UFH dose increase to a mean of 21 IU/kg/h lead to therapeutic Anti-Xa values (0.50–0.69 IU/ml) to which a further increase of the CT IN/HI ratio of 2.268 correlated. In contrast, mean aPTT remained at 52 s without depicting UFH dose increase of increased Anti-Xa activity.

Within the category Anti-Xa ≥ 0.70 IU/ml, mean UFH dose was 23 IU/kg/h and the corresponding mean values for aPTT and CT IN/HI ratio were 55 s and 2.726, respectively. Of note,with an upper limit of normal (ULN) of 36 s for aPTT in our laboratorya mean aPTT of 55 s correspondended to an aPTT ratio (aPTT/ULN) of 1.53 despite a supratherapeutic Anti-Xa activity of ≥ 0.70 IU/ml. 

### Diagnostic performance of aPTT and CT IN/HI ratio

**Fig. 2:**
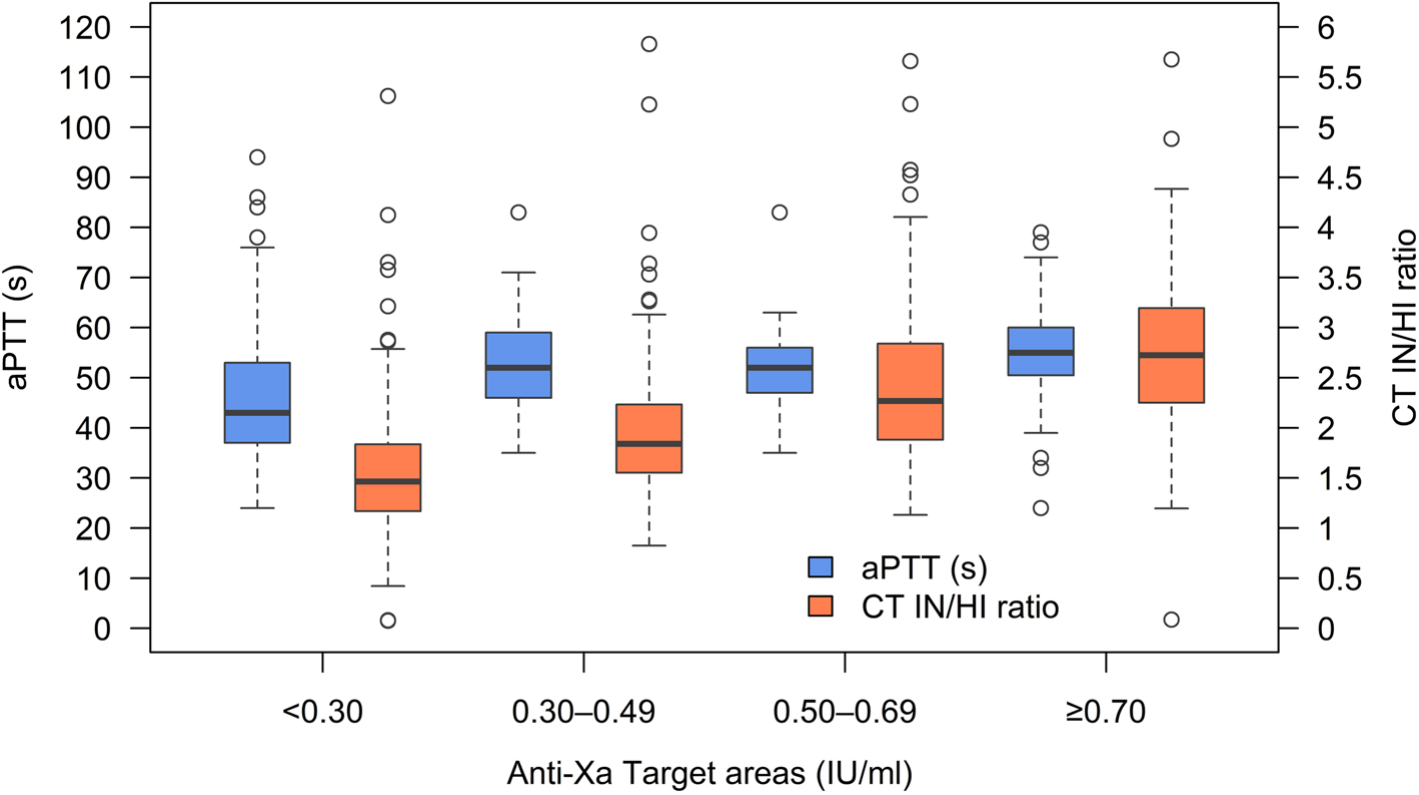
Distribution of aPTT and CT IN/HI ratio values across Anti-Xa activity ranges. Boxplots of aPTT (blue, left y-axis) and CT IN/HI ratio (orange, right y-axis) stratified by Anti-Xa activity categories (< 0.30 IU/mL, 0.30–0.49 IU/mL, 0.50–0.69 IU/mL, ≥ 0.70 IU/mL; x-axis). Boxes represent the interquartile range (IQR) with median values shown as horizontal lines, whiskers indicate 1.5 × IQR, and circles denote outliers. The figure illustrates the increasing trend of both aPTT and CT IN/HI ratio with rising Anti-Xa activity. aPTT, activated partial thromboplastin time

**Fig. 3:**
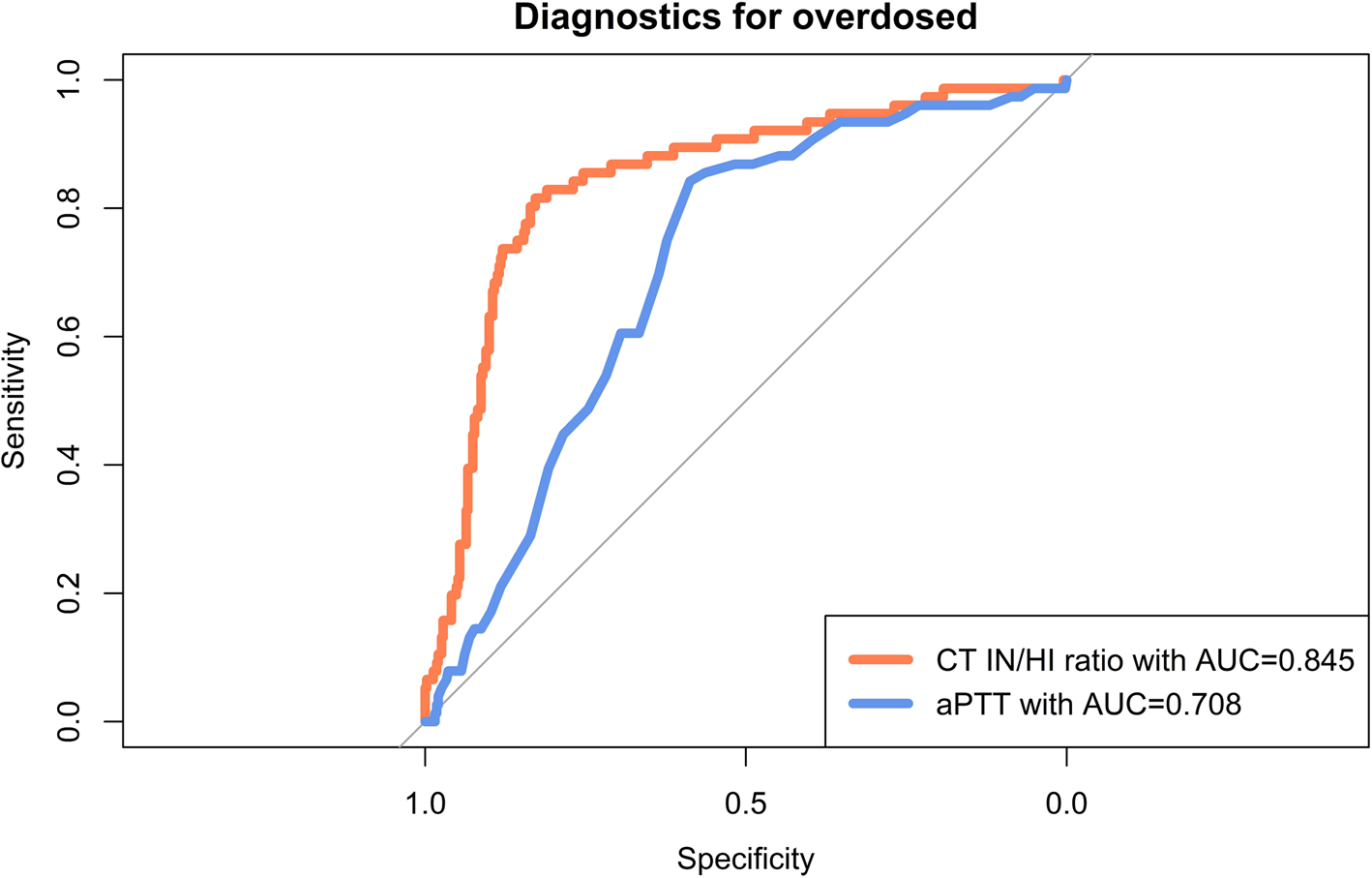
ROC analysis for detection of overdosing. ROC comparing the diagnostic performance of the CT IN/HI ratio (orange) and aPTT (blue) for identifying Anti-Xa overdosing with Anti-Xa ≥ 0.70 IU/ml. The CT IN/HI ratio demonstrated superior discrimination with an AUC of 0.845 compared to an AUC of 0.708 for aPTT. The diagonal line represents the line of no discrimination. aPTT, activated partial thromboplastin time

Figure 2 exhibits the distributions of the test results of aPTT and CT IN/HI ratio for each Anti-Xa category. Compared to aPTT the CT IN/HI ratio values increased more strongly for each category of Anti-Xa (Fig. 2, Table 2). The discriminatory performance of aPTT and the CT IN/HI ratio across different Anti-Xa activity ranges was evaluated using ROC curves. For the prophylactic and overdose analyses, all values outside the respective reference group were included. For the therapeutic analysis, samples classified as therapeutic or sub-therapeutic based on Anti-Xa levels were considered. For the detection of supratherapeutic Anti-Xa levels, the aPTT yielded an AUC of 0.708, the CT IN/HI ratio demonstrated an AUC of 0.845. The CT IN/HI ratio outperformed aPTT in both detection of therapeutic ranges and overdosing (Table 3, Fig. 3, Supplemental Digital Content 2). For the identification of prophylactic Anti-Xa levels, both assays showed lower discriminatory performance compared with the higher Anti-Xa ranges; however, the CT IN/HI ratio still yielded a higher AUC (0.817) than aPTT (0.726) (Supplemental Digital Content 1). For the discrimination between therapeutic and subtherapeutic levels the AUC for aPTT was 0.494, while the CT IN/HI ratio showed discrimination with an AUC of 0.690 (Supplemental Digital Content 2). In addition, the number of paired measurements was sufficient to allow robust comparison of diagnostic performance and threshold estimation. Differences between AUCs were formally assessed using DeLong’s test, which demonstrated statistically significant superiority of the CT IN/HI ratio over aPTT for all three predefined comparisons (prophylactic, therapeutic, and supratherapeutic ranges; *p* = 3.65 × 10⁻^5^, *p* = 0.00118, and *p* = 1.43 × 10⁻⁷, respectively).

**Table 3:** Confusion matrix for the weighted Cohen’s kappa (equal spacing) for four categories of Anti-Xa und aPTT and the CT IN/HI ratio for 466 measurements

Clinical classification of Anti-Xa test results	Prophylactic	Sub-therapeutic	Therapeutic	Overdosing
aPTT [s]				
< 50	182	35	12	12
50–59	50	30	23	44
60–79	27	20	5	20
80 +	4	1	1	0
CT IN/HI ratio				
< 1.413	125	10	3	4
1.413–1.916	89	39	8	6
1.917–2.332	36	20	13	10
> 2.332	13	17	17	56

Confusion matrix showing the distribution of 466 paired measurements across four predefined clinical categories of Anti-Xa activity (prophylactic, sub-therapeutic, therapeutic, overdosing) compared with corresponding results for aPTT (top) and the CT IN/HI ratio (bottom). Values represent counts of observations within each cell. Weighted Cohen’s kappa (equal spacing) was applied to assess agreement between the clinical classification of Anti-Xa test results and the two alternative monitoring parameters

### Threshold estimation and multiclass classification

Threshold estimation for the CT IN/HI ratio yielded values of 1.413–1.916, 1.917–2.332, and > 2.332 for the identification of sub-therapeutic, therapeutic, and overdose Anti-Xa ranges, respectively. The corresponding sensitivities for these thresholds were 47.5%, 73.2%, and 73.7%, while the specificities were 91.6%, 57.0%, and 87.9%, respectively. The positive predictive values (PPVs), or precisions, were 0.88, 0.45, and 0.54.

The aPTT thresholds for the prophylactic, sub-therapeutic, and therapeutic ranges were 50 s, 60 s, and 80 s, respectively, in accordance with institutional standards. The sensitivities corresponding to these thresholds in the sample were 71.1%, 14.6% and 0%. The corresponding specificities were 63.1%, 75.6% and 98.5%, respectively. The positive predictive values were 0.71, 0.22 and 0.

When these thresholds are used in a confusion matrix (Table 3), we estimate the alignment of the aPTT and the CT IN/HI ratio to the reference Anti-Xa test, with a Cohens kappa coefficient of 0.450 (95% 0.390–0.509), which is moderate according to Landis et al. (1977). For aPTT, the Cohen’s kappa is 0.220 (95% 0.164–0.276), which is considered borderline to no alignment [38].

## Discussion

In this prospective observational study, we assessed the diagnostic performance of the CT IN/HI ratio derived from viscoelastic point-of-care testing compared with aPTT for monitoring UFH in critically ill patients, using Anti-Xa activity as the reference standard. The CT IN/HI ratio demonstrated superior diagnostic accuracy across anticoagulation ranges, particularly for distinguishing therapeutic from subtherapeutic UFH levels, where aPTT showed limited discriminatory capacity. Exploratory threshold analyses identified clinically usable CT IN/HI cut-offs with favorable specificity and moderate agreement with Anti-Xa. However, the modest positive predictive values, especially in therapeutic and supratherapeutic ranges, indicate that the CT IN/HI ratio is influenced by coagulation factors beyond UFH alone. Consequently, while the CT IN/HI ratio may sensitively identify potentially excessive anticoagulation, confirmation by Anti-Xa testing remains necessary when precise quantification of heparin activity is required.

Although Anti-Xa remains the analytical reference standard for UFH monitoring, limited availability and turnaround time restrict real-time decision-making. The CT IN/HI ratio provides immediate bedside results and demonstrated stronger diagnostic alignment with Anti-Xa than aPTT in both binary and multiclass analyses. However, as no clinical endpoints were assessed, no conclusions regarding safety or patient outcomes can be drawn. The potential advantages of VET must therefore be balanced against the wider availability and lower cost of aPTT, and routine VET-based UFH monitoring cannot be justified without evidence of clinical benefit.

### Comparison of aPTT to Anti-Xa

Our findings align with existing studies that highlight the limitations of aPTT as a monitoring tool for UFH. In this study, aPTT exhibited a lower AUC than did the CT IN/HI ratio in differentiating therapeutic anticoagulation ranges compared with Anti-Xa levels. For example, none of the 76 overdosed measurements were identified by aPTT, indicating significant overlap between therapeutic and nontherapeutic ranges. This limitation has been corroborated by studies showing substantial variability between aPTT and Anti-Xa levels. Takemoto et al. (2013) reported concordance between heparin levels measured by Anti-Xa assay and aPTT in only 54% of measurements, highlighting the discordance between these two methods [25]. Similarly, a 2020 study by Ratano et al. revealed that 40% of paired observations contained therapeutic ranges of aPTT and Anti-Xa [39]. Other studies revealed similar results, underscoring the inconsistency of aPTT as a reliable marker of anticoagulation [22, 23, 40].

In contrast, Anti-Xa levels have consistently demonstrated greater reliability in achieving and maintaining therapeutic anticoagulation. Whitman-Purves et al. reported that Anti-Xa assays provided more consistent results, reducing the risk of subtherapeutic or supratherapeutic dosing with improved time to therapeutic anticoagulation and fewer dose adjustments [41]. Similarly, Zaki et al. reported that Anti-Xa-guided dosing achieved therapeutic targets more rapidly and maintained them more consistently than did aPTT-guided dosing [42]. Additionally, specific to ECMO patients, studies have revealed that the accordance between aPTT and Anti-Xa measurement is consistently low [24, 43].

The aPTT thresholds applied in this analysis were not defined specifically for this study but reflect institutional standard operating procedures for UFH monitoring based on commonly used clinical practice ranges. These thresholds were intentionally used to evaluate the performance of aPTT under real-world conditions rather than to derive optimized study-specific cut-offs. Consequently, discrepancies between aPTT-based classification and Anti-Xa–defined anticoagulation ranges likely reflect the well-described limitations of aPTT as a surrogate marker of heparin activity in critically ill patients.

### Point-of-care testing with different devices

The feasibility of using the CT IN/HI ratio for UFH monitoring at the POC offers significant advantages over laboratory-based methods such as Anti-Xa assays. The CT IN/HI ratio, as measured by VET devices such as ROTEM**®**, ClotPro**®**, and TEG®, provides real-time data on coagulation dynamics, enabling immediate clinical decision-making. This study demonstrated that the CT IN/HI ratio was strongly aligned with Anti-Xa levels, with a Cohen’s kappa of 0.450, whereas the aPTT was 0.220. Furthermore, the ability of the CT IN/HI ratio to distinguish between therapeutic, prophylactic, and overdosed anticoagulation states emphasizes its clinical utility. Nevertheless, VET devices also present important drawbacks. Cartridge costs are considerably higher than for standard coagulation assays, and device downtime or calibration issues can limit continuous availability. Moreover, variability between platforms (ROTEM®, ClotPro®, TEG®) complicates standardization of UFH-specific thresholds across institutions. These factors represent practical barriers to widespread implementation of VET for routine UFH monitoring.

Moreover, VET offers several advantages over ACT, which is why ACT was not included in the present work. Supporting this rationale, Ichikawa et al. demonstrated that both ACT and aPTT failed to detect residual heparin following protamine reversal, whereas a ROTEM®-based clotting time (CT) ratio of two assays (INTEM/HEPTEM) showed a markedly superior correlation with circulating heparin concentrations as measured by a chromogenic Anti-Xa assay [44]. These findings indicate that (i) ACT tends to underestimate anticoagulant activity at low heparin levels—a scenario frequently encountered in ICU patients under prophylactic or subtherapeutic UFH therapy—and (ii) VET may constitute a promising alternative class of point-of-care devices for UFH monitoring.

ROTEM**®** and ClotPro**®** have been extensively studied for UFH monitoring. Yin et al. (2024) compared the use of TEG® and ROTEM**®** for monitoring UFH in patients undergoing ECMO [45]. They reported that the intrinsic clotting time (INTEM CT) in ROTEM**®** was less sensitive to lower doses of UFH, suggesting that TEG® might be preferable for monitoring anticoagulation in this subgroup. Additionally, Dias et al. (2019) assessed the feasibility of the cartridge-based TEG®6 s system for quantifying the effects of UFH and LMWH [33]. Their results demonstrated that TEG®6 s accurately classified heparin activity into subtherapeutic, therapeutic, and supratherapeutic ranges, achieving an accuracy of over 90% for UFH. However, neither study evaluated their devices against the gold standard Anti-Xa levels, which is a unique strength of this study.

Katz et al. reported a moderate correlation between the INTEM/HEPTEM coagulation time ratio and plasma heparin concentration in an exploratory in vitro obstetric model, supporting the conceptual feasibility of viscoelastic ratios for UFH assessment. However, the lack of clinical validation limits direct applicability to routine practice. In contrast, the present prospective study in critically ill patients directly compared viscoelastic ratios with Anti-Xa activity, providing stronger evidence for the clinical relevance of the CT IN/HI ratio [46].

The CT IN/HI ratio compares intrinsic clotting times with and without heparinase, providing a UFH-specific measure that improves sensitivity over intrinsic clotting time alone. By neutralising heparin, this ratio reduces variability from non–heparin-related factors and allows more precise UFH assessment. Consistent with this concept, the CT IN/HI ratio showed strong alignment with Anti-Xa activity in our study, whereas prior work has reported limited sensitivity of intrinsic clotting times at lower UFH doses [45]. The use of the CT IN/HI ratio in ClotPro**®** uniquely bridges this gap by integrating UFH-specific monitoring with broader coagulation assessments.

Furthermore, the rapid turnaround time and POC applicability of VET`s make it particularly advantageous in dynamic clinical settings. By integrating real-time data with threshold-based monitoring, ClotPro**®** enables clinicians to adjust UFH dosing promptly, reducing the risk of complications. However, Haemonetics© recently officially announced the discontinuation of the ClotPro® platform, which was used in our study [38]. However, with the introduction of the successor system Multiclot*®* by apiro, including continued availability of heparin-specific assays, the concept of viscoelastic monitoring of anticoagulation remains of high clinical relevance[47]. This ensures that, despite changes in device availability, the methodological principle and clinical applicability of VET-based UFH monitoring will persist and continue to evolve.

### Optimal thresholds for different indications

Determining optimal thresholds for different ranges of anticoagulation is critical for balancing efficacy and safety in UFH therapy. Thresholds were estimated strictly for exploratory diagnostic purposes to illustrate how the CT IN/HI ratio distributes across Anti-Xa-defined categories in this cohort. These thresholds are not intended to guide clinical decision-making, and prospective validation is required before any clinical application. This study identified CT IN/HI ratio thresholds that align with Anti-Xa-defined ranges:

- Prophylactic anticoagulation: CT IN/HI ratio < 1.413
- Sub-therapeutic anticoagulation: CT IN/HI ratio 1.413–1.916
- Therapeutic anticoagulation: CT IN/HI ratio 1.917–2.332
- Avoiding Overdosing: CT IN/HI ratio > 2.332

At a CT IN/HI threshold of 2.332, sensitivity and specificity for detecting UFH overdosing were 0.737 and 0.879, respectively, with a PPV of 0.544. In contrast, aPTT showed substantial overlap between therapeutic and supratherapeutic ranges, limiting its dosing precision. Notably, overlap between Anti-Xa categories was also observed: 18.6% of measurements classified as prophylactic by Anti-Xa exhibited CT IN/HI values above 1.917. These findings indicate that CT IN/HI thresholds are exploratory and cannot be used to infer specific UFH dosing intensities, underscoring the need for external validation and prospective interventional studies before clinical implementation. The ability to stratify anticoagulation states is particularly important in critically ill patients, where the risks of thromboembolic events and bleeding are increased. Vo et al. emphasized the importance of avoiding overdosing, as supratherapeutic anticoagulation is associated with increased bleeding complications [43]. Despite the expectation of an increased bleeding risk at supratherapeutic Anti-Xa levels, only four bleeding events were observed in this range. This distribution is consistent with previous reports indicating a limited predictive value of Anti-Xa activity for bleeding events, although conflicting findings have been described. Heterogeneity of the critically ill population and the temporal dissociation between Anti-Xa measurements and bleeding events likely contributed to the observed variability.

### Study limitations

This study has several limitations that should be acknowledged. First, the relatively small number of patients included limits the generalizability of the findings to broader populations. Furthermore the majority of the patients were on ECMO which further limits the applicability for our findings to a general audience. Although 466 measurements were available from these patients. Larger cohorts are necessary to validate the thresholds and performance metrics identified for the CT IN/HI ratio. Second, the observational design of the study may have introduced confounding factors that were not fully accounted for, such as variations in ATIII levels and fibrinogen concentrations, which are known to influence the CT IN/HI ratio and aPTT. Third, the study population was predominantly composed of critically ill patients. While this provides valuable insights into this high-risk group, the findings may not be directly applicable to noncritically ill populations or patients with other clinical conditions requiring UFH. Additionally, the reliance on specific devices and assays for the CT IN/HI ratio and Anti-Xa measurements may affect the reproducibility of the results in different contexts. Potential sex-specific differences or in different batches in heparin response cannot be excluded and represent a limitation of this work. Finally, the measurements for adverse events are collected in proximity to the event, not at the exact event time. A significant limitation of monitoring UFH via Anti-Xa measurement is that it neglects UFH`s direct anticoagulant effects on thrombin (Factor II). Given that UFH typically has stable effects on FX and FII, an increasing Anti-II effect may also be observed with Anti-Xa monitoring. Another challenge is that Anti-Xa testing tends to be costly, time-consuming, and may not be readily available 24/7 [48].

Beyond these methodological limitations, important practical considerations must also be addressed before routine implementation of VET-based UFH monitoring can be recommended. Compared with aPTT, VET devices are associated with higher acquisition and per-test costs, require specifically trained non-laboratory personnel, and are not widely available in many ICUs. Routine VET-guided anticoagulation management would therefore increase healthcare expenditures unless a clear clinical benefit is demonstrated. As this study did not assess clinical outcomes, no conclusions regarding bleeding or thrombotic event reduction can be drawn.

## Conclusion

This study highlights the advantages of the CT IN/HI ratio as a POC test for UFH monitoring in critically ill patients. Compared with aPTT, the CT IN/HI ratio demonstrates superioralignment with Anti-Xa levels and greater specificity in identifying therapeutic anticoagulation ranges for defined thresholds. While Anti-Xa activity served as the analytical reference standard in this study, the CT IN/HI ratio may represent a useful alternative in situations where rapid bedside assessment is required or Anti-Xa testing is not readily available. The direct comparison with Anti-Xa underscores the diagnostic performance of the CT IN/HI ratio within the limitations of this study. However, implementation on a broader scale must consider higher costs, limited availability, and the lack of evidence for improved patient outcomes. Future studies should focus on validating device-specific thresholds, assessing cost-effectiveness, and determining whether VET-guided UFH adjustments translate into measurable improvements in clinical outcomes.

## Supplementary Information

Supplementary Material 1: ROC analysis for detection of prophylactic range. ROC curves comparing the ability of the CT IN/HI ratio (orange) and aPTT (blue) to identify Anti-Xa levels in the prophylactic range, i.e. Anti-Xa <0.30 IU/ml. The CT IN/HI ratio showed superior discrimination with an AUC of 0.817 compared to an AUC of 0.726 for aPTT. The diagonal line indicates the line of no discrimination. aPTT, activated partial thromboplastin time.

Supplementary Material 2: ROC analysis for therapeutic versus sub-therapeutic classification. ROC curves for aPTT (blue) and CT IN/HI ratio (orange) in differentiating therapeutic from sub-therapeutic Anti-Xa levels. Only measurements with Anti-Xa between 0.30 IU/mL and 0.70 IU/mL (*n* = 129) were included. The CT IN/HI ratio demonstrated moderate discriminatory ability for detection of Anti-Xa between 0.50-0.70 IU/ml with an AUC of 0.690, whereas aPTT showed no relevant discrimination with an AUC of 0.494. The diagonal line represents the line of no discrimination. aPTT, activated partial thromboplastin time.

## Data Availability

The datasets generated and/or analysed during the current study are not publicly available due to data protection and privacy regulations but are available from the corresponding author on reasonable request.

## Abbreviations

ACT: Activated clotting time
Anti-Xa: Anti-factor Xa activity
aPTT: Activated partial thromboplastin time
ATIII: Antithrombin III
AUC: Area under the curve
BARC: Bleeding Academic Research Consortium
BMI: Body-Mass-Index
CFT: Clot formation time
COVID-19: Coronavirus disease 2019
CT: Clotting time
DVT: Deep vein thrombosis
ECMO: Extracorporeal membrane oxygenation
EX-test: Extrinsic test
HI-test: Heparinase-test
ICU: Intensive Care Unit
IN-test: Intrinsic-test
IQR: Interquartile range
LMWH: Low molecular weight heparin
LT: Lysis time
MCF: Maximum clot firmness
ML: Maximum lysis
POC: Point-of-care
PPV: Positive predictive value
ROC: Receiver operating characteristic
ROTEM **®**: Rotational thromboelastometry **®**
SOFA: Sequential organ failure assessment
SOP: Standard operating procedure
TE: Thromboembolic event
TEG™: Thromboelastography™
UFH: Unfractionated heparin
VET: Viscoelastic testing
