# Supplementary figures and images for: Viscoelastic point-of-care testing for monitoring unfractionated heparin in critically ill patients: a prospective observational study comparing ClotPro® IN/HI-Test, aPTT and Anti-Xa activity
Source: BMC Anesthesiol. 2026 Mar 24;26:222. doi: 10.1186/s12871-026-03781-4 (PMC13063743; doi:10.1186/s12871-026-03781-4)

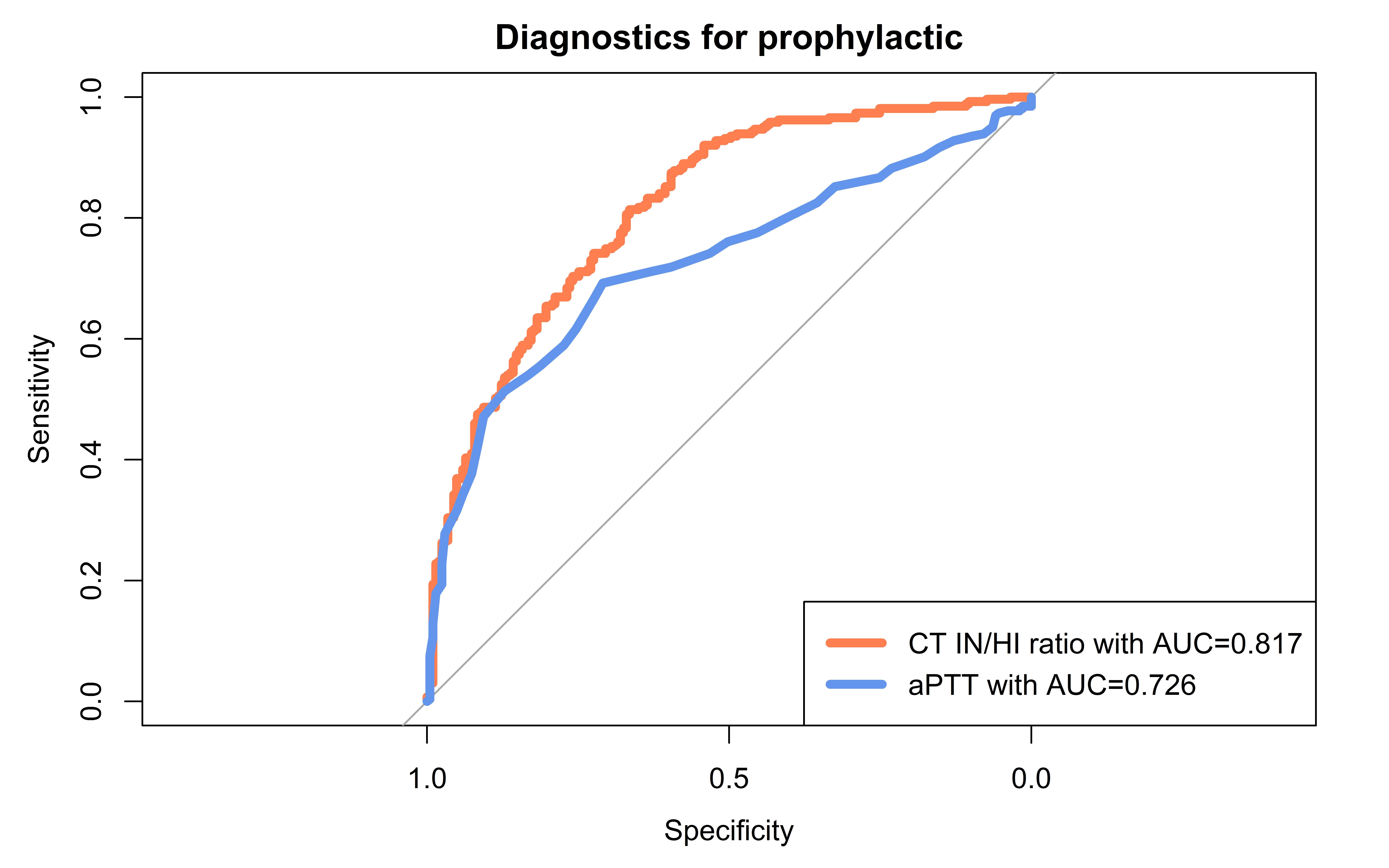

Supplement: Supplementary file 1 — Supplementary Material 1: ROC analysis for detection of prophylactic range. ROC curves comparing the ability of the CT IN/HI ratio (orange) and aPTT (blue) to identify Anti-Xa levels in the prophylactic range, i.e. Anti-Xa <0.30 IU/ml. The CT IN/HI ratio showed superior discrimination with an AUC of 0.817 compared to an AUC of 0.726 for aPTT. The diagonal line indicates the line of no discrimination. aPTT, activated partial thromboplastin time. [file 12871_2026_3781_MOESM1_ESM.jpg]

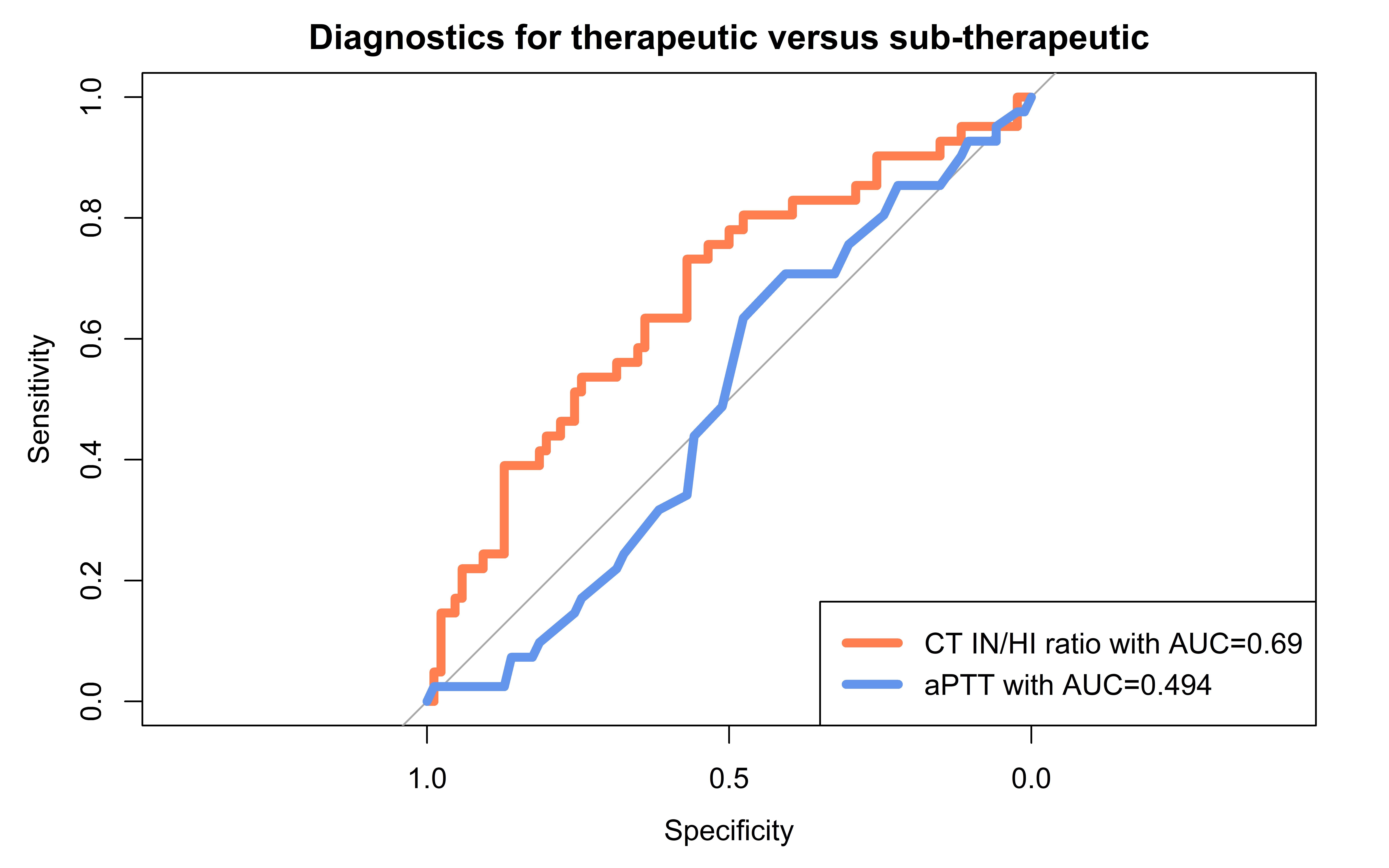

Supplement: Supplementary file 2 — Supplementary Material 2: ROC analysis for therapeutic versus sub-therapeutic classification. ROC curves for aPTT (blue) and CT IN/HI ratio (orange) in differentiating therapeutic from sub-therapeutic Anti-Xa levels. Only measurements with Anti-Xa between 0.30 IU/mL and 0.70 IU/mL (n = 129) were included. The CT IN/HI ratio demonstrated moderate discriminatory ability for detection of Anti-Xa between 0.50-0.70 IU/ml with an AUC of 0.690, whereas aPTT showed no relevant discrimination with an AUC of 0.494. The diagonal line represents the line of no discrimination. aPTT, activated partial thromboplastin time. [file 12871_2026_3781_MOESM2_ESM.jpg]
